# Meta-analysis of maternal and neonatal outcomes of cannabis use in pregnancy current to March 2024

**DOI:** 10.1186/s40748-025-00216-9

**Published:** 2025-08-01

**Authors:** Katelyn Sainz, Hollie Ulibarri, Amanda Arroyo, Daniela Gonzalez Herrera, Brooke Hamilton, Kate Ruffley, McKenna Robinson, Greg J. Marchand

**Affiliations:** 1 Dartmouth Health Children’s, Department of Neonatology, Lebanon, NH USA; 2 Marchand Institute for Minimally Invasive Surgery, Mesa, AZ USA

**Keywords:** Pregnancy, Cannabis, Marijuana Smoking, Neonatal Outcomes, Newborn, Maternal Exposure

## Abstract

**Importance:**

Following expansive legalization of cannabis in many parts of the United States, cannabis use in pregnancy has increased several fold. There is a pressing need to understand the maternal and neonatal outcomes associated with this exposure.

**Objective:**

To quantify the maternal and neonatal outcomes of mothers using cannabis during pregnancy.

**Data sources:**

We searched five databases for all relevant observational studies, from each database’s inception until March 1st 2024.

**Study selection:**

Two reviewers separately screened the studies in duplicate. Our initial search yielded 5184 studies, of which 51 (0.98%) were included in our qualitative synthesis.

**Data extraction and synthesis:**

Our study adhered to PRISMA guidelines and independent extraction by two researchers was utilized. We used a 95% confidence interval and the random effects model, as there was significant heterogeneity between studies.

**Results:**

The 51 included studies yielded a total population of 7,920,383 pregnant women. Cannabis consumption was associated with increased risks of low birth weight (RR = 1.69,95% CI = (1.34,2.14)*,P* < 0.0001), small for gestational age (RR = 1.79,95% CI = (1.52, 2.1),*P* < 0.00001), major anomalies (RR = 1.81,95% CI = (1.48, 2.23),*P* < 0.00001), decreased head circumference (MD = -0.34,95% CI = (-0.57,-0.11),*P* = 0.004), birth weight (MD = -177.81,95% CI = (-224.72,-130.91),*P* < 0.00001), birth length (MD = -0.87,95% CI = (-1.15,-0.59),*P* < 0.00001), gestational age (MD = -0.21,95% CI = (-0.35,-0.08),*P* = 0.002), NICU admission (RR = 1.55,95% CI = (1.36,1.78),*P* < 0.00001), perinatal mortality (RR = 1.72,95% CI = (1.09,2.71),*P* = 0.02), and preterm delivery (RR = 1.39,95% CI = (1.23,1.56),*P* < 0.00001). Cannabis use was also associated with a decreased risk of gestational diabetes in pregnancy (RR = 0.64,95% CI = (0.55,0.75),*P* < 0.00001).

**Conclusions:**

Inclusion of the latest published data continues to show worse maternal and neonatal outcomes for mothers using cannabis in pregnancy.

## Introduction

The daily consumption of cannabis is increasing in the United States from 3% in 2002 to 7% in 2017 to 11% today [1]. Rates are even higher in reproductive age adults with teens at 22% and young adults at 19% [2]. The best estimates of consumption during pregnancy reach approximately 4.5% [3], making cannabis the most common illegal substance used during pregnancy [4]. Over half of women using cannabis prior to pregnancy choose to continue use during pregnancy, especially during the first trimester which includes fetal organogenesis [5, 6].

One possible cause for this increase may be the legalization of medical and recreational cannabis in many regions of the United States [7]. This has the potential to increase the perception among pregnant women that cannabis use may be safe or that it could represent a lower risk alternative to other medications during pregnancy [8, 9]. This comes despite most major obstetrical organizations continuing to encourage discontinuation in women who are or plan to become pregnant [10, 11].

Fetal effects of cannabis are theorized to occur secondary to delta-9-tetrahydrocannabinol (THC), which crosses the placenta and binds to receptors present on fetal cells [12]. THC binding to the cannabinoid receptors may result in disruption of cannabinoid signaling, which may then result in alterations of levels of dopamine, GABA, serotonin, adrenalin, and glutamate; potentially interfering with placental and/or fetal development [13, 14].

Despite recommendations, the harmful effects of cannabis during pregnancy are still controversial, and recent meta-analyses are not in complete agreement. A link for even the most commonly associated outcome, low birth weight [15, 16], has not been found in all meta-analyses [17]. Other outcomes, such as increased maternal hypertension [16, 18], increased rates of preterm delivery [18], increased neonatal invasive care unit (NICU) admission [15, 16], increased infant death rates [19], and maternal psychological disorders [20, 21], are inconsistently found to be associated with cannabis in different meta-analyses.

In an attempt to solve this controversy, we aimed to conduct the largest systematic review and meta-analysis performed thus far, including all possible observational studies in order to obtain the largest sample size.

## Methods

Our systematic review and meta-analysis was reported according to the Preferred Reporting Items for Systematic Reviews and Meta-analysis (PRISMA) [22].

### Searching databases

We performed our search through all major databases, including Web of Science, PubMed, Cochrane Library, ClinicalTrials.Gov and SCOPUS. We used the following search strategy ("Pregnancy"OR"Pregnant Women"OR pregnant OR pregnancy OR Gestation) AND ("Cannabis"OR Ganjas OR Hemps OR Hashish OR Hashishs OR Bhang OR Bhangs OR cannabis OR Cannabis OR marihuana OR ganja OR Hemp OR weed OR hash OR"Mary Jane") for all relevant articles from each database’s inception until March 1 st 2024.

### Inclusion and exclusion criteria

The inclusion criteria used were (1) population of pregnant females; (2) exposure of cannabis use of any frequency or method of reporting; (3) comparison was cannabis non-users; (4) outcomes were maternal and neonatal outcomes; and (5) study design included any double armed observational studies (such as prospective cohort studies, retrospective cohort studies, cross-sectional studies, or case–control studies.)

The exclusion criteria were non-pregnant women, single-arm studies, case reports, case series, studies published in languages rather than English, reviews, conference abstracts, editorial letters or notes, and animal studies.

### Screening and study selection

The resulting records from searching databases were exported into EndNote X8.0.1 [23] which were then exported to Excel software after removing duplicates to start screening which was done independently by screening title and abstracts according to the inclusion criteria. Then, the full texts of the resulting records were screened also to determine the final included studies. Any conflict about the inclusion of any article was solved by consensus between the authors.

### Data extraction

First, we extracted general demographics from the included studies. This included the study name, country, design, study dates, the number of participants in each group, the method of determining cannabis use, maternal age in each group, alcohol use in each group, number of smokers in each group, and number of women older than 35 years. Next, we extracted the maternal and neonatal outcomes in each group, which included the maternal outcomes (gestational diabetes mellitus, preeclampsia, cesarean section, and gestational hypertension) and the neonatal outcomes (low birth weight (defined as less than 2500 g), small for gestational age (defined as less than the 10 th percentile), preterm delivery before 37 weeks, NICU admission, birth weight in grams, the perinatal mortality rate (defined as the percentage of fetal deaths in pregnancies of seven or more months plus number of deaths of live-born children in the first 6 days following birth), gestational age, birth length in centimeters, head circumference in centimeters, major and minor congenital anomalies, major anomalies, and gender.)

### Quality assessment

The quality assessment was performed using the Newcastle Ottawa Scale. This is a star-based method composed of three main items: selection of each group, group comparability, and exposure ascertainment [24]. Each study was assessed and a total score was given to determine the final judgment of whether the study was of poor (0–3 stars), fair (4–6 stars), or good quality (7–9 stars) [24].

### Statistical analysis

We performed this analysis with Review Manager Software using a risk ratio (RR) with a 95% confidence interval (CI) for the qualitative variables and mean difference (MD) with a 95% CI for the quantitative variables. The heterogeneity between studies in each outcome was assessed using the I^2^ statistical test and Cochrane Q test. The outcomes were considered heterogeneous when the I^2^ was > 50% and the *P* value was < 0.1 [25]. The random effects model was chosen due to the presented heterogeneity between the included studies. We tried to solve the presented heterogeneity by the “leave-one-out"method, to exclude the study responsible for causing heterogeneity [25]. Results were considered significant when the determined *P* values were below 0.05. Given the potential influence of confounding variables like smoking, we relied on the random-effects model to incorporate between-study differences, including variations in adjustment for confounders. While smoking status data were extracted where available (Table 2), we did not perform subgroup analyses based on adjustment for smoking due to inconsistent reporting across studies and the lack of uniform covariate adjustment data, which would limit the reliability of such stratification.

## Results

### Literature search results

**Fig. 1:**
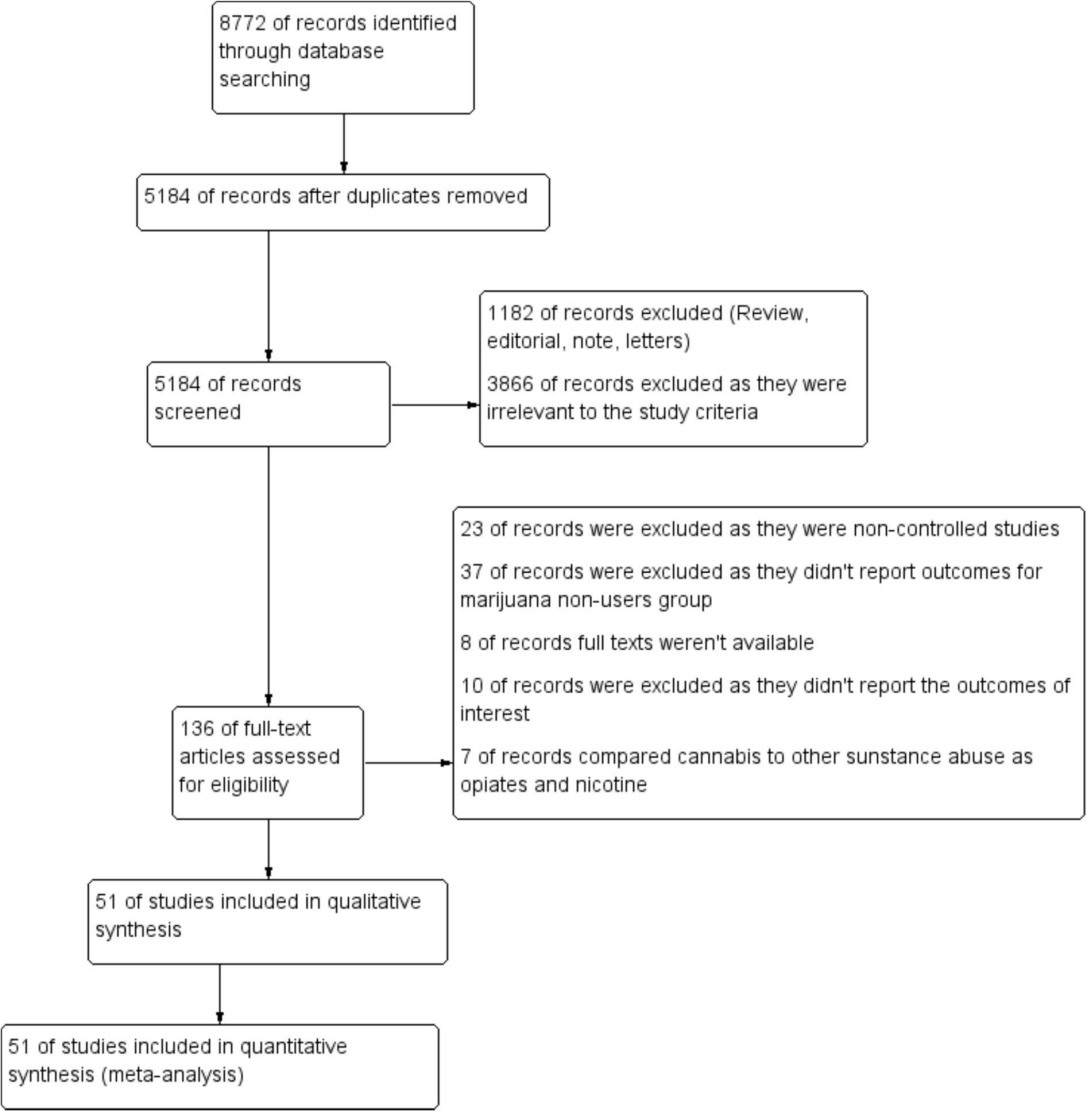
Prisma diagram of our study search and selection process

The literature search resulted in 5184 studies after removing duplicates, all of which entered the title and abstract screening phase. From there, only 136 were eligible for the next phase, which was full-text screening. This ultimately resulted in 51 studies being eligible to be included in the meta-analysis. Figure 1 shows the PRISMA flow diagram explaining the full details of screening results and the study selection process.

### General demographic data of the included results

We included 51 observational studies with a total population of 7,920,383 women 111,939 were cannabis users and 7,808,444 were non-users [21, 26–75]. Twenty-seven studies were retrospective cohort studies [21, 27, 29–31, 37, 42, 45, 48, 49, 51, 52, 55–57, 60, 62–65, 67–71, 73, 75], 22 studies were prospective cohort studies [26, 28, 32–36, 38–41, 43, 44, 46, 47, 50, 54, 58, 61, 66, 72, 74], one study was cross-sectional [59], and one was case–control study [53]. Tables 1 and 2 show the full details of the general demographic data of the included studies.

**Table 1:** General demographic data of the included studies

**Author **	Country	Study Design	Study Dates	Marijuana user group (number)	Non-Marijuana users group (number)	Method of determining Marijuana Use
Avalos et al., 2023 [37]	United States	retrospective cohort	Between January 1, 2011, and July 31, 2020	22,624	342,300	Self-reported and urine toxicology screening
Dodge et al., 2023 [29]	United States	retrospective cohort	Between 2016 and 2020	109	171	Self-reported urine toxicology screening or cord toxicology screening
Dunn et al., 2023 [31]	Australia	retrospective cohort	Between January 1, 2019 and December 31, 2019	50	3054	Self-reported
Prewitt et al., 2023 [55]	United States	retrospective cohort	Between 2007 and 2011	9,144	2,371,302	Self-reported
Jones et al., 2022 [68]	Canada	retrospective cohort	Between January 1, 2017 and June 20, 2019	483	1057	Meconium toxicology screening
Koto et al., 2022 [49]	Canada	retrospective cohort	Between January 1, 2004 and June 30, 2004	3144	103 138	Self-reported
Metz et al., 2022 [67]	United States	retrospective cohort	Not reported	47	980	Urine toxicology
Brik et al., 2022 [70]	Spain	retrospective cohort	Between January 2013 and December 2020	60	198	Urine toxicology
Bruno et al., 2022 [72]	United States	prospective cohort	Between October 2010 and September 2013	136	9027	Self-reported and urine toxicology screening
Luke et al., 2022 [52]	Canada	retrospective cohort	Between April 1, 2012 and March 31, 2019	20410	1031360	Self-reported
Klebanoff et al., 2020 [46]	United States	prospective cohort	Between 2010 and 2016	117	244	Urine toxicology
Gabrhelik et al., 2021 [36]	Norway	prospective cohort	Between 1999 and 2008	272	10101	Self-reported
Bandoli et al., 2021 [48]	United States	retrospective cohort	Between 2011 and 2017	29112	3037957	Diagnostic code
Sasso et al., 2021 [57]	United States	retrospective cohort	Between 2014 and 2018	151	192	Self-reported
Straub et al., 2021 [69]	United States	retrospective cohort	Between March 11, 2011 and March 31, 2016	1268	4075	Urine toxicology
Bailey et al., 2020 [71]	United States	retrospective cohort	Not reported	531	531	Urine toxicology
Grzeskowiak et al., 2020 [40]	New Zealand, United Kingdom, Australia and Ireland	prospective cohort	Between November 2004 and February 2011	217	5393	Self-reported
Kharbanda et al., 2020 [45]	United States	retrospective cohort	Between July 1, 2015, and December 1, 2017	283	3152	Urine toxicology
Klebanoff et al., 2021 [47]	United States	prospective cohort	Between 2010 and 2015	119	244	Self-reported and urine toxicology screening
Nawa et al., 2020 [53]	United States	case-control	Between 1998 and 2018	328	5933	Self-reported
Corsi et al., 2019 [27]	Canada	retrospective cohort	Between April 1, 2012, and December 31, 2017	9427	652190	Self-reported
Luke et al., 2019 [51]	Canada	retrospective cohort	Between April 1, 2008 and March 31, 2016	5801	237339	Self-reported
Rodriguez et al., 2019 [56]	United States	retrospective cohort	Between September 2011 and May 2017	211	995	Self-reported and urine toxicology screening
Ko et al., 2018 [63]	United States	retrospective cohort	Between 2012 and 2015	463	8549	Self-reported
Coleman-Cowger et al., 2018 [74]	United States	prospective cohort	Between January and December 2017	60	354	Self-reported and urine toxicology screening
Serino et al., 2018 [58]	United States	prospective cohort	Between 2004 and 2010	38	49	Self-reported
Dotters-Katz et al., 2017 [30]	United States	retrospective cohort	Between 1997 and 2004	135	1732	Self-reported and urine toxicology screening
Metz et al., 2017 [21]	United States	retrospective cohort	Between March 2006 and September 2008	48	1562	Self-reported and THC-COOH (11-Nor-9-carboxy-THC) detection in umbilical cord homogenate
Leemaqz et al., 2016 [50]	New Zealand, United Kingdom, Australia and Ireland	prospective cohort	Between November 2004 and February 2011	315	95	Self-reported
Mark et al., 2016 [64]	United States	retrospective cohort	Between July 1, 2009 and June 30, 2010	116	280	Self-reported and urine toxicology screening
Warshak et al., 2015 [62]	United States	retrospective cohort	Between January 2008 and January 2011	361	6107	Self-reported and urine toxicology screening
Conner et al., 2016 [15]	United States	retrospective cohort	Between 2004 and 2008	680	7458	Self-reported and urine toxicology screening
Alhusen et al., 2013 [26]	United States	prospective cohort	Between February 2009 and February 2010	64	102	Self-reported
Hayatbakhsh et al., 2012 [42]	Australia	retrospective cohort	Between 2000 and 2006	647	24227	Self-reported
Gray et al., 2010 [39]	United States	prospective cohort	Not reported	38	48	Self-reported, meconium toxicology screening and oral fluid toxicology screening
El Marroun et al., 2010 [33]	Netherlands	prospective cohort	Between April 2002 and January 2006	23	85	Self-reported
El Marroun et al., 2009 [32]	Netherlands	prospective cohort	Between April 2002 and January 2006	214	5785	Self-reported
Burns et al., 2006 [73]	Australia	retrospective cohort	Between 1998 and 2002	2172	412 731	Diagnostic code
Barros et al., 2006 [59]	Brazil	cross-sectional	Not reported	26	534	Maternal hair and neonatal meconium
Hurd et al., 2005 [44]	United States	prospective cohort	Between January 2000 and December 2002	44	95	Self-reported, urine toxicology screening and neonatal meconium screening
Fergusson et al., 2002 [34]	England	prospective cohort	Between April 1, 1991 and December 31, 1992	250	11890	Self-reported
Sherwood et al., 1999 [60]	United Kingdom	retrospective cohort	Between November 1994 and May 1995	75	213	Urine toxicology
Parker et al., 1999 [54]	United States	prospective cohort	Between July, 1984 through June, 1987	202	1024	Urine toxicology
Day et al., 1991 [28]	United States	prospective cohort	Not reported	174	210	Self-reported
Witter et al., 1990 [65]	United States	retrospective cohort	Between 1983 and 1985	417	7933	Self-reported
Zuckerman et al., 1989 [66]	United States	prospective cohort	Between July 1984 and June 1987	202	895	Self-reported and urine toxicology screening
Hayes et al., 1988 [43]	Jamaica	prospective cohort	Not reported	30	26	Self-reported
Hatch et al., 1987 [41]	United States	prospective cohort	Between May 12, 1980, and March 12, 1982	367	3490	Self-reported
Tennes et al., 1985 [61]	United States	prospective cohort	Between November 1981 and November 1982	258	498	Self-reported
Fried et al., 1984 [35]	Canada	prospective cohort	Not reported	84	499	Self-reported
Gibson et al., 1983 [38]	Australia	prospective cohort	Not reported	392	6909	Self-reported

**Table 2:** General demographic data of the included studies

**Study name**	Maternal age						Alcohol abuse				Smoking				Maternal age ≥ 35			
	MJ users			MJ non-users			MJ users		MJ non-users		MJ users		MJ non-users		MJ users		MJ non-users	
Author	mean	SD	total	mean	SD	total	event	total	event	total	event	total	event	total	event	total	event	total
Avalos et al., 2023 [37]	-	-	-	-	-	-	4335	22624	28524	342300	5566	22624	12255	342300	-	-	-	-
Dodge et al., 2023 [29]	22.7	3.6	109	25.4	4.8	171	-	-	-	-	-	-	-	-	-	-	-	-
Dunn et al., 2023 [31]	-	-	-	-	-	-	8	50	103	3054	33	50	222	3054	3	50	735	3054
Prewitt et al., 2023 [55]	-	-	-	-	-	-	412	9144	2075	2371302	2382	9144	71206	2371302	636	9144	411808	2371302
Jones et al., 2022 [68]	26.5	5.1	483	27.6	5.7	1057	12	483	23	1057	214	483	398	1057	-	-	-	-
Koto et al., 2022 [49]	25.7	5.39	3144	29.8	5.5	103138	226	3144	206	103138	1886	3144	16502	103138	-	-	-	-
Metz et al., 2022 [67]	-	-	-	-	-	-	-	-	-	-	24	47	137	980	1	47	137	980
Brik et al., 2022 [70]	28.5	5.21	60	30.7	4.2	198	0	60	0	198	0	60	0	198	-	-	-	-
Bruno et al., 2022 [72]	22.9	4.4	136	26.5	5.82	9027	-	-	-	-	71	136	550	9027	-	-	-	-
Luke et al., 2022 [52]	-	-	-	-	-	-	2768	20410	17417	1031360	11232	20410	80379	1031360	1688	20410	231860	1031360
Klebanoff et al., 2020 [46]	25.8	5.1	116	26.7	5.4	243	34	117	45	244	79	117	78	244	-	-	-	-
Gabrhelik et al., 2021 [36]	-	-	-	-	-	-	212	265	6921	9918	108	204	1516	7831	29	271	1356	10045
Bandoli et al., 2021 [48]	-	-	-	-	-	-	1499	29112	4732	3037957	10721	29112	82645	3037957	-	-	-	-
Sasso et al., 2021 [57]	27.62	6.19	151	30.2	7.12	192					64	151	10	192	-	-	-	-
Straub et al., 2021 [69]	25.85	5.28	1268	27.04	5.72	4075	356	1268	1089	4075	1025	1268	2126	4075	-	-	-	-
Bailey et al., 2020 [71]	24.4	5.3	531	24.4	5.1	531	11	531	11	531	353	531	353	531	-	-	-	-
Grzeskowiak et al., 2020 [40]	23.8	5.7	217	28.86	5.42	5393	30	217	540	5393	111	217	486	5393	-	-	-	-
Kharbanda et al., 2020 [45]	25.4	5.3	283	29.9	5	3152	-	-	-	-	118	283	186	3152	-	-	-	-
Klebanoff et al., 2021 [47]	-	-	-	-	-	-	30	119	44	244	49	119	77	244	-	-	-	-
Nawa et al., 2020 [53]	-	-	-	-	-	-	-	-	-	-	-	-	-	-	-	-	-	-
Corsi et al., 2019 [27]	-	-	-	-	-	-	1787	9427	13185	652190	5554	9427	48260	652190	435	9427	110208	652190
Luke et al., 2019 [51]	-	-	-	-	-	-	700	5801	2168	237339	4038	5801	39370	237 339	451	5801	55517	237339
Rodriguez et al., 2019 [56]	18.8	1.5	211	18.8	1.8	995	0	211	4	995	45	211	47	995	-	-	-	-
Ko et al., 2018 [63]	-	-	-	-	-	-	79	463	667	8549	199	463	1060	8549	45	463	1334	8549
Coleman-Cowger et al., 2018 [74]	27.3	4.9	60	28.2	5.4	354	17	60	61	354	0	60	0	354	-	-	-	-
Serino et al., 2018 [58]	23.9	4.9	38	25.9	5.6	49	-	-	-	-	-	-	-	-	-	-	-	-
Dotters-Katz et al., 2017 [30]	26.33	6.74	135	25.67	6.68	1732	-	-	-	-	104	135	431	1732	-	-	-	-
Metz et al., 2017 [21]	-	-	-	-	-	-	-	-	-	-	28	48	183	1562	2	48	217	1562
Leemaqz et al., 2016 [50]	-	-	-	-	-	-	-	-	-	-	-	-	-	-	-	-	-	-
Mark et al., 2016 [64]	22.9	5	116	23	5.9	280	8	116	6	280	50	116	53	280	-	-	-	-
Warshak et al., 2015 [62]	24	5.2	361	25.3	5.9	6107	-	-	-	-	208	361	1214	6107	-	-	-	-
Conner et al., 2016 [15]	24	5.3	680	25	6.1	7458	52	680	60	7458	395	680	1066	7458	-	-	-	-
Alhusen et al., 2013 [26]	-	-	-	-	-	-	-	-	-	-	-	-	-	-	-	-	-	-
Hayatbakhsh et al., 2012 [42]	-	-	-	-	-	-	-	-	-	-	-	-	-	-	-	-	-	-
Gray et al., 2010 [39]	24.4	5.1	38	24.3	5.2	48	-	-	-	-	33	38	22	48	-	-	-	-
El Marroun et al., 2010 [33]	29.35	3.86	23	31.8	3.7	85	13	23	40	85	19	23	0	85	-	-	-	-
El Marroun et al., 2009 [32]	26.76	5.76	214	29.99	5.16	5785	41	214	902	5785	116	214	77	5785	-	-	-	-
Burns et al., 2006 [73]	-	-	-	-	-	-	88	2172	0	412731	1679	2172	67487	412731	-	-	-	-
Barros et al., 2006 [59]	16.5	1.5	26	16.9	1.5	534	-	-	-	-	-	-	-	-	-	-	-	-
Hurd et al., 2005 [44]	22.4	0.6	44	23.4	0.7	95	24	44	22	95	17	44	17	95	-	-	-	-
Fergusson et al., 2002 [34]	25.5		250	27.8		11890	86.5	250	2306	11890	172	250	2794	11890	-	-	-	-
Sherwood et al., 1999 [60]	-	-	-	-	-	-	-	-	-	-	-	-	-	-	-	-	-	-
Parker et al., 1999 [54]	-	-	-	-	-	-	-	-	-	-	162	202	307	1024	-	-	-	-
Day et al., 1991 [28]	22.91	-	174	22.9	-	210	130	174	109	210	122	174	86	210	-	-	-	-
Witter et al., 1990 [65]	-	-	-	-	-	-	222	417	2499	7933	327	417	2975	7933	-	-	-	-
Zuckerman et al., 1989 [66]	24	4.9	202	24.1	5.7	895	-	-	-	-	-	-	-	-	-	-	-	-
Hayes et al., 1988 [43]	-	-	-	-	-	-	-	-	-	-	-	-	-	-	-	-	-	-
Hatch et al., 1987 [41]	-	-	-	-	-	-	303	367	2364	3490	211	367	978	3490	3	367	201	3490
Tennes et al., 1985 [61]	21.8	-	258	23	-	498	181	258	149	498	103	258	149	498	-	-	-	-
Fried et al., 1984 [35]	26.1	-	84	29.3	-	499	5	84	15	499	15	84	25	499	-	-	-	-
Gibson et al., 1983 [38]	-	-	-	-	-	-	-	-	-	-	-	-	-	-	-	-	-	-

### Results of the quality assessment

According to the Newcastle Ottawa scale, the majority of the included cohorts were judged to be of fair quality [76]. They showed a low risk of bias in the outcome assessment and comparability domains. However, in some studies the method of determining exposure was based on self-reports, the analysis was not controlled for confounders, and several studies did not specifically report the outcomes of interest. Notably, Hayes et al., Hayatbakhsh et al., Alhusen et al., and Leemaqz et al. were judged to be of poor quality because of these factors [26, 42, 43, 50]. Likewise, Zuckerman et al., Sherwood et al., and Dodge et al. were also judged to be of poor quality, despite using more scientific methods to determine cannabis exposure [29, 60, 66]. Witter et al., Hurd et al., Burns et al., Conner et al., Mark et al., Metz et al., Jones et al., and Avalos et al. were judged to be of good quality since they showed a low risk of bias in selection, comparability, and outcome assessment domains [21, 37, 44, 64, 65, 68, 73, 75]. Nawa et al. is the only included case–control study and it was judged to be of poor quality since their analysis was not controlled for confounders. Moreover, their exposure determination was also based on self-reporting [53].

Barros et al. was the only included cross-sectional study. It was judged as good quality since there was no risk of bias in the three domains of the Newcastle Ottawa scale [59]. Table 3 shows the full details of the quality assessment results.

**Table 3:** Quality assessment of the included cohort studies

		Selection				Comparability	Outcome			Quality Judgment
Author	year	Representativeness of the exposed cohort	Selection of the non exposed cohort	Ascertainment of exposure	Demonstration that outcome of interest was not present at start of study	Comparability of cohorts on the basis of the design or analysis	Assessment of outcome	Was follow-up long enough for outcomes to occur	Adequacy of follow up of cohorts	
Avalos et al.	2023	*	*	*		**	*	*	*	good
Dodge et al.	2023	*	*	*			*	*	*	poor
Dunn et al.	2023	*	*			**	*	*	*	fair
Prewitt et al.	2023	*	*			**	*	*	*	fair
Jones et al.	2022	*	*	*	*	**	*	*	*	good
Koto et al.	2022	*	*			**	*	*	*	fair
Metz et al.	2022	*	*	*		*	*	*	*	fair
Brik et al.	2022	*	*	*		**	*	*	*	good
Bruno et al.	2022	*	*	*	*	*	*	*	*	good
Luke et al.	2022	*	*			**	*	*	*	fair
Klebanoff et al.	2020	*	*	*		**	*	*	*	good
Gabrhelik et al.	2021	*	*			**	*	*	*	fair
Bandoli et al.	2021	*	*	*		**	*	*	*	good
Sasso et al.	2021	*	*			*	*	*	*	fair
Straub et al.	2021	*	*			**	*	*	*	fair
Bailey et al.	2020	*	*			**	*	*	*	fair
Grzeskowiak et al.	2020	*	*			**	*	*	*	fair
Kharbanda et al.	2020	*	*	*		*	*	*	*	fair
Klebanoff et al.	2021	*	*	*		**	*	*	*	good
Corsi et al.	2019	*	*			**	*	*	*	fair
Luke et al.	2019	*	*			**	*	*	*	fair
Rodriguez et al.	2019	*	*	*	*	**	*	*	*	good
Ko et al.	2018	*	*			**	*	*	*	fair
Coleman-Cowger et al.	2018	*	*	*	*	**	*	*	*	good
Serino et al.	2018	*	*			**	*	*	*	fair
Dotters-Katz et al.	2017	*	*	*		*	*	*	*	fair
Metz et al.	2017	*	*	*	*	*	*	*	*	good
Leemaqz et al.	2016	*	*				*	*	*	poor
Mark et al.	2016	*	*	*		**	*	*	*	good
Warshak et al.	2015	*	*	*		*	*	*	*	fair
Conner et al.	2016	*	*	*		**	*	*	*	good
Alhusen et al.	2013	*	*				*	*	*	poor
Hayatbakhsh et al.	2012	*	*				*	*	*	poor
Gray et al.	2010	*	*	*		*	*	*	*	fair
El Marroun et al.	2010	*	*			**	*	*	*	fair
El Marroun et al.	2009	*	*			**	*	*	*	fair
Burns et al.	2006	*	*	*		**	*	*	*	good
Hurd et al.	2005	*	*	*		**	*	*	*	good
Fergusson et al.	2002	*	*			**	*	*	*	fair
Sherwood et al.	1999	*	*	*			*	*	*	poor
Parker et al.	1999	*	*	*		*	*	*	*	fair
Day et al.	1991	*	*			**	*	*	*	fair
Witter et al.	1990	*	*	*	*	**	*	*	*	good
Zuckerman et al.	1989	*	*	*			*	*	*	poor
Hayes et al.	1988	*	*				*	*	*	poor
Hatch et al.	1987	*	*			**	*	*	*	fair
Tennes et al.	1985	*	*			**	*	*	*	fair
Fried et al.	1984	*	*			**	*	*	*	fair
Gibson et al.	1983	*	*			**	*	*	*	fair

### Maternal outcomes

**Fig. 2:**
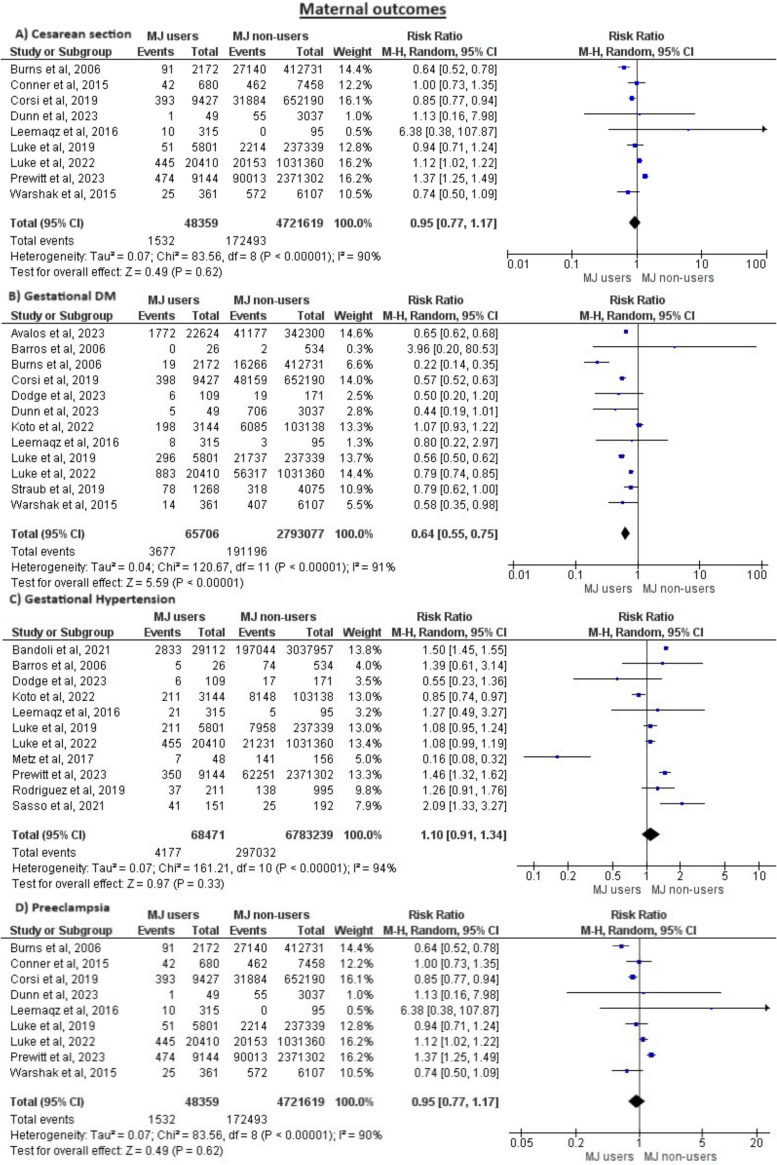
Meta-analysis of all maternal outcomes

We compared the following maternal outcomes between both groups: cesarean section, gestational diabetes, gestational hypertension, and preeclampsia; however, all these outcomes showed no significant differences between the groups except for gestational diabetes which was significantly decreased in cannabis users compared to non-users (RR = 0.64, 95% CI = (0.55, 0.75), *P* < 0.00001). However, this outcome was heterogeneous (like most other maternal outcomes) and we could not solve heterogeneity by leave-one-out method (*P* < 0.00001, I^2^ = 91%), Fig. 2 shows the analysis of maternal outcomes.

### Neonatal outcomes

**Fig. 3:**
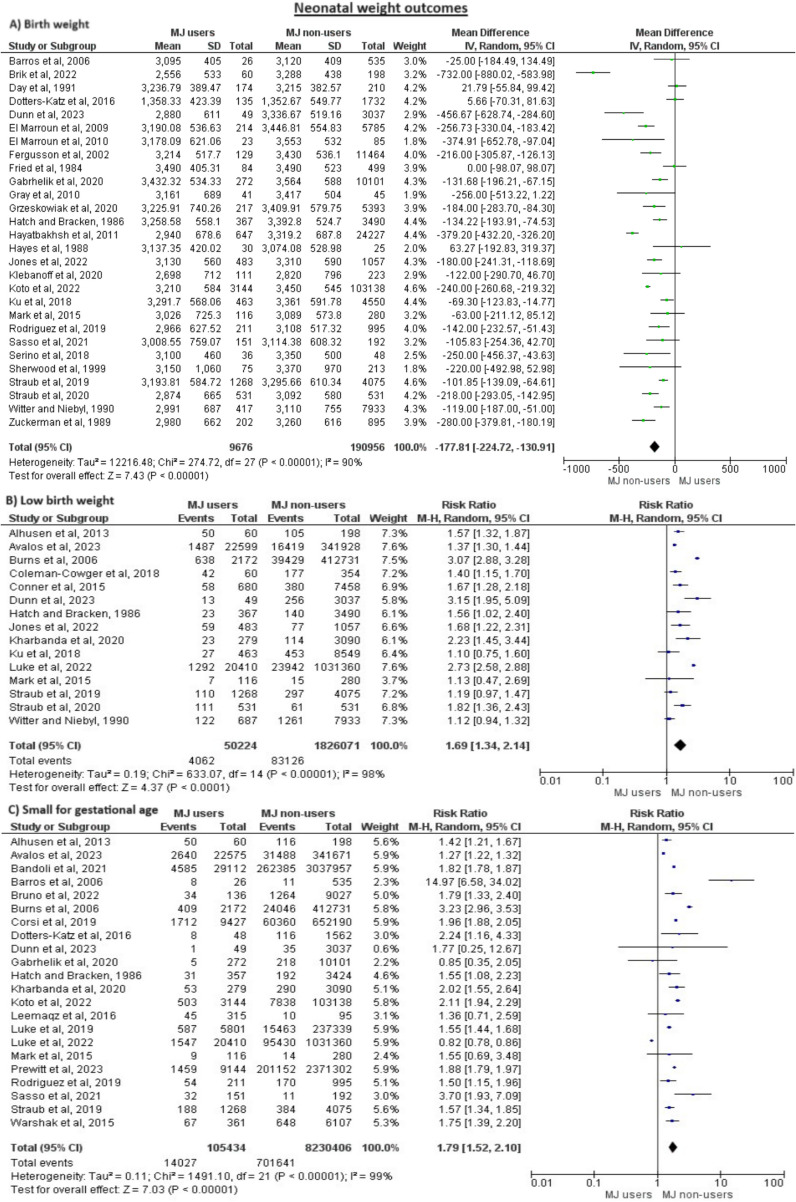
Meta-analysis of neonatal outcomes related to birth weight

Regarding neonatal weight outcomes including the birth weight, the incidence of low birth weight, and the diagnosis of small for gestational age, all of these showed results that favored the non-user group as there was decreased birth weights in cannabis users (MD = −177.81, 95% CI = (−224.72, −130.91), *P* < 0.00001), an increased number of low birth weight infants (RR = 1.69, 95% CI = (1.34, 2.14), *P* < 0.0001), and an increased number of infants diagnosed as small for gestational age (RR = 1.79, 95% CI = (1.52, 2.1), *P* < 0.00001). However, all these outcomes were again heterogeneous and we could not solve the heterogeneity using any method. Figure 3 shows the full details.

**Fig. 4:**
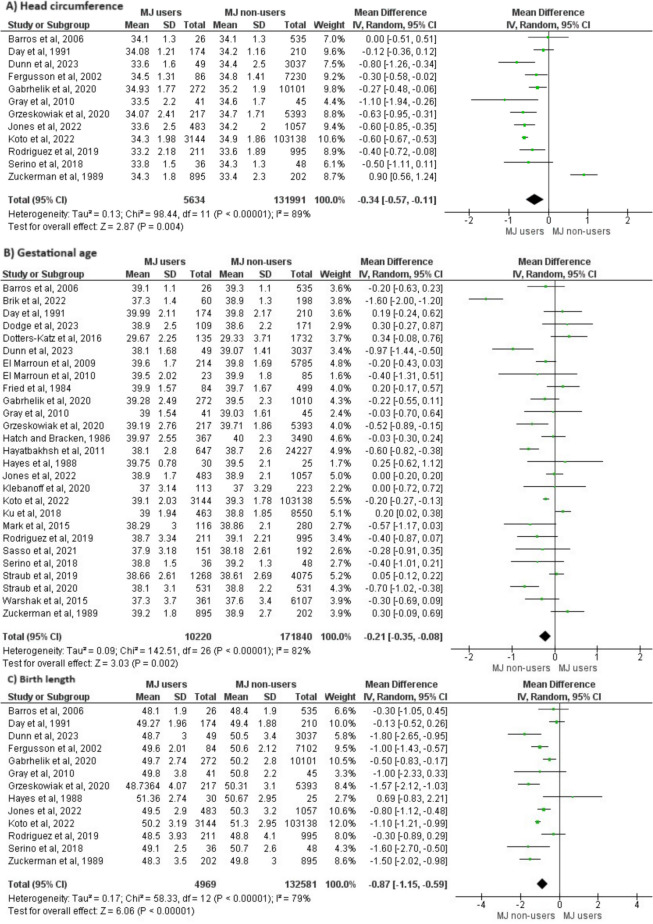
Meta-analysis of neonatal head circumference, gestational age, and birth length

Regarding other neonatal characteristics, head circumference, gestational age, and birth length were also significantly decreased in cannabis users compared to non-users (MD = −0.34, 95% CI = (−0.57, −0.11), *P* = 0.004), (MD = −0.21, 95% CI = (−0.35, −0.08), *P* = 0.002), and (MD = −0.87, 95% CI = (−1.15, −0.59), *P* < 0.00001), respectively. Again, all these outcomes were heterogeneous and we could not solve the heterogeneity. Figure 4 shows the full details.

**Fig. 5:**
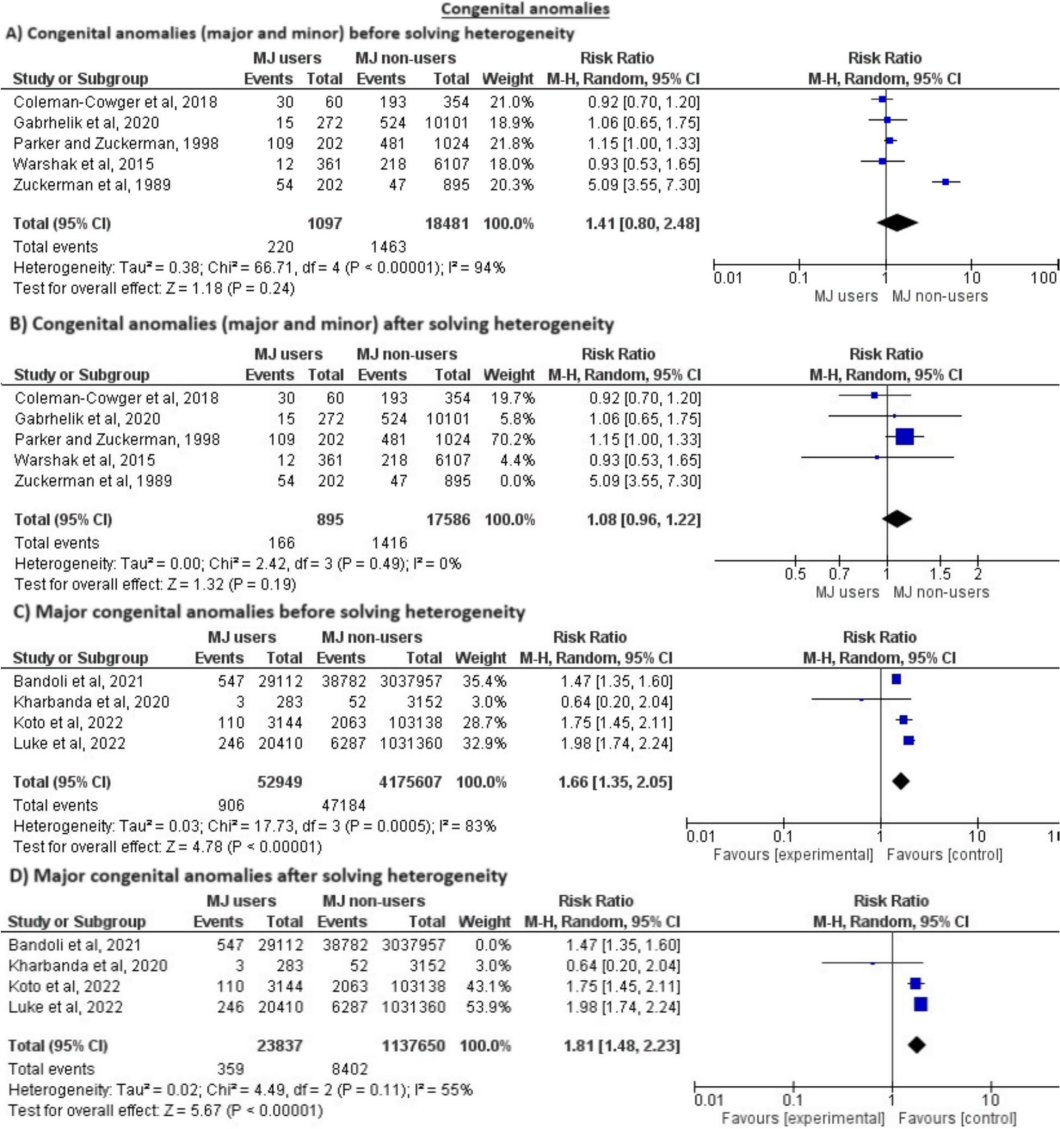
Meta-analysis of the incidence of congenital abnormalities

Regarding anomalies, the combination of major and minor anomalies showed no significant difference between the two groups, but was also heterogeneous. In order to solve the heterogeneity, we excluded Zuckerman 1989 et al. [66] from the analysis, however the outcome still did not reach statistical significance (RR = 1.08, 95% CI = (0.96, 1.22), *P* = 0.19) and (*P* = 0.49, I^2^ = 0%), as seen in Fig. 5.

There was an increased risk of only major anomalies in cannabis users compared to non-users; however, the outcome was heterogeneous. This was solved by excluding Bandoli 2021 et al. [48] and the results remained significant (RR = 1.81, 95% CI = (1.48, 2.23), *P* < 0.00001) and (*P* = 0.11, I^2^ = 55%), as seen in Fig. 5.

**Fig. 6:**
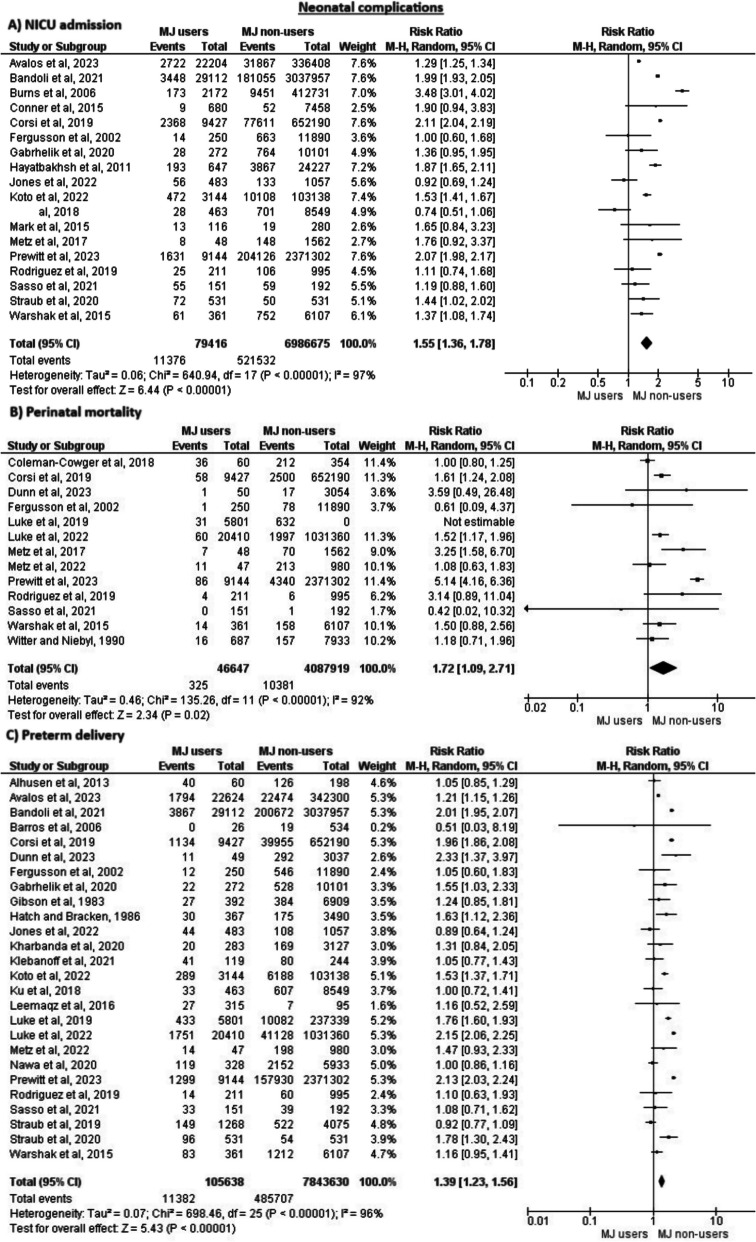
Meta-analysis of the incidence of NICU admission, perinatal mortality and preterm delivery

Also, complications like NICU admission, perinatal mortality, and preterm delivery were significantly decreased among cannabis non-users compared to users (RR = 1.55, 95% CI = (1.36, 1.78), *P* < 0.00001), (RR = 1.72, 95% CI = (1.09, 2.71), *P* = 0.02), and (RR = 1.39, 95% CI = (1.23, 1.56), *P* < 0.00001), respectively. However, all these outcomes were heterogeneous and none could be solved by recognized methods, as seen in Fig. 6.

**Fig. 7:**
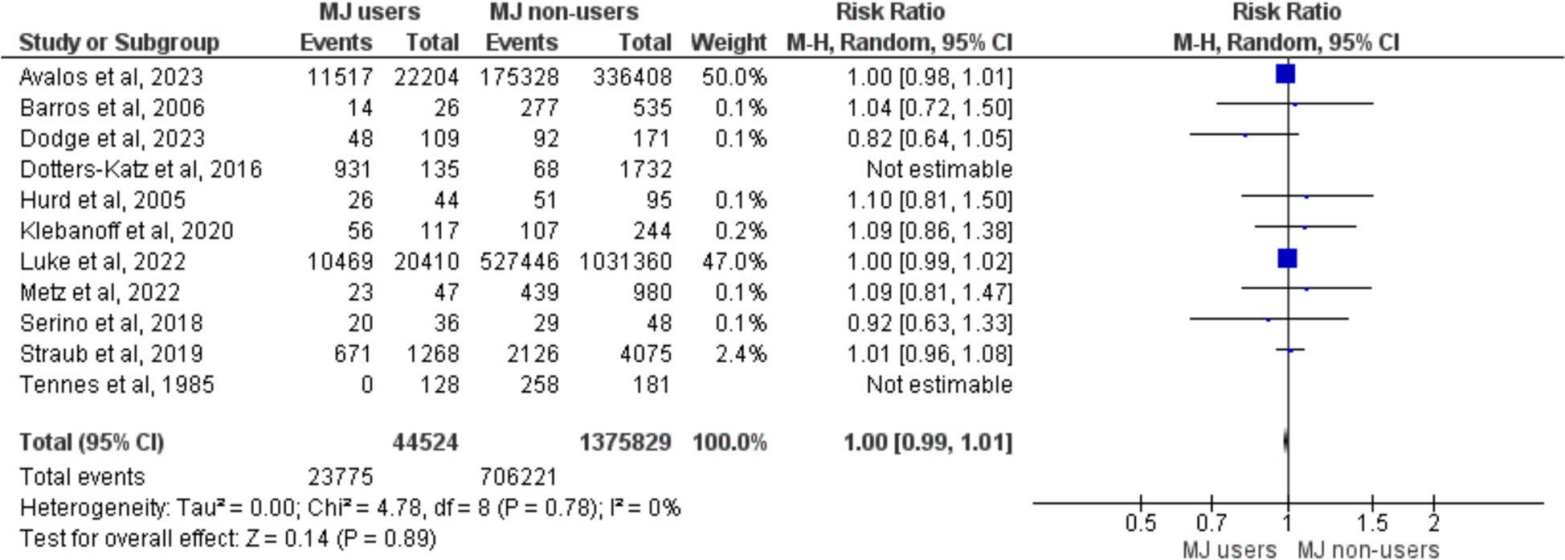
Meta-analysis of fetal gender

As expected, cannabis use had no effect on infant gender (RR = 1, 95, 95% CI = (0.99, 1.01), *P* = 0.89), as seen in Fig. 7.

## Discussion

Our systematic review included 7,920,383 women and found that cannabis consumption was associated with increased risks of low birth weight, small for gestational age, major anomalies, decreased head circumference, decreased neonatal weight, decreased birth length, decreased gestational age, NICU admission, perinatal mortality, and preterm delivery; however, it was associated with decreased risk of gestational diabetes. This constitutes the largest meta-analysis on this subject to date, and hopefully will add strong evidence to the argument that cannabis use in pregnancy is associated with poor neonatal outcomes. As stated below, however, many questions still remain unanswered as far as if these findings apply to all methods of ingesting cannabis, and if these results remain relevant when controlling for tobacco smoking, environmental exposures, and alcohol use in pregnancy. As for the unexpected finding of an association between cannabis use and reduced gestational diabetes risk, our researchers speculate that this may be the result of the common practice of using cannabis to alleviate chronic joint pain from morbid obesity. Many of these individuals likely have already been diagnosed with Type II diabetes prior to pregnancy, thus making it impossible for them to receive a diagnosis of gestational diabetes, and giving the misleading impression that cannabis may protect against the same. This hypothesis requires further investigation due to limited data on pregestational diabetes prevalence.

We acknowledge the significant heterogeneity observed across most outcomes, which is not unexpected given the inclusion of 51 studies spanning diverse populations, methodologies, and exposure definitions. Potential sources of this heterogeneity include variations in the frequency, quantity, and recency of cannabis use, which our binary classification (users vs. non-users) may not fully capture. For instance, heavy or frequent use might amplify adverse outcomes compared to occasional use, while recency, such as use concentrated in the first trimester versus throughout pregnancy, could influence fetal development differently due to critical windows of organogenesis. Additionally, the method of assessing cannabis exposure varied across studies, with some relying on self-reports and others using biological validation (e.g., urine toxicology or meconium screening), as detailed in Table 1. These differences could contribute to heterogeneity by affecting the accuracy and consistency of exposure classification. For example, self-reports may underestimate use due to social desirability bias, whereas biological measures might detect use that participants did not disclose.

Many systematic reviews and meta-analyses have supported the effect of cannabis consumption in increasing risks of neonatal adverse effects, especially preterm delivery, NICU admission, low birth weight, and smaller head circumference, as was seen in our findings [16, 18, 77].

Our meta-analysis, encompassing 51 studies and 7,920,383 women, aligns with and extends findings from prior meta-analyses by Conner 2016 et al. [15], Gunn 2016 et al. [16], Lo 2023 et al. [17], and Marchand 2022 et al. [18]. Like Gunn 2016 and Marchand 2022, we found significant associations between prenatal cannabis use and increased risks of low birth weight (RR = 1.69, 95% CI = 1.34–2.14 vs. Gunn’s OR = 1.77 and Marchand’s OR = 1.87), preterm delivery (RR = 1.39, 95% CI = 1.23–1.56 vs. Gunn’s OR = 1.43, Lo’s elevated risk, and Marchand’s OR = 1.42), SGA (RR = 1.79, 95% CI = 1.52–2.1, consistent with Lo and Marchand), and NICU admission (RR = 1.55, 95% CI = 1.36–1.78, echoing Gunn’s OR = 2.02 and Marchand’s findings). However, our results diverge from Conner 2016, which reported no independent cannabis effect after adjusting for tobacco (OR = 1.43 for low birth weight reduced post-adjustment), suggesting our broader, unadjusted associations may partly reflect confounding. Unlike Gunn’s unique finding of maternal anemia (OR = 1.36), we found no significant maternal outcomes except a decreased gestational diabetes risk (RR = 0.64, 95% CI = 0.55–0.75), potentially a spurious signal. Compared to Lo 2023, which found no clear cannabis-only mortality link, our increased perinatal mortality (RR = 1.72, 95% CI = 1.09–2.71) suggests newer studies may amplify this signal, though with borderline significance. Our inclusion of 35 additional studies beyond Marchand 2022’s 16 reinforces these associations, adding novel outcomes like major anomalies (RR = 1.81, 95% CI = 1.48–2.23) and decreased head circumference (MD = −0.34, 95% CI = −0.57 to −0.11), not emphasized in earlier works. This expanded scope, current to March 2024, suggests a consistent pattern of neonatal risk, though confounding remains a challenge, aligning with all four prior reviews’ cautions.

Associated smoking with cannabis consumption could be an important confounding factor that can be responsible for this association as found in Conner 2016 et al. who found that there was no significant difference between cannabis users and non-users regarding neonatal outcomes after controlling confounders like tobacco smoking [15] which was supported also by English 1997 et al. [78] who included only studies which adjusted the tobacco use. This effect results from the larger percentage of cannabis smokers also smoking cigarettes during pregnancy than non-users [79]. While our large sample size (over 7 million women) suggests robustness, uncontrolled tobacco use remains a potential confounder, as noted in prior studies [15, 78]. Further evidence for this has been presented in the 2017 cross-sectional analysis by Haight et al. [80], which found high frequency cannabis use was related to lower birth weights regardless of cigarette use. To further explore this, we reviewed the 51 included studies and found that approximately 20 (39%) explicitly reported adjusting for smoking status in their statistical analyses (e.g., Conner et al., 2015; Metz et al., 2017; Avalos et al., 2023), as noted in their respective methodologies or results sections [15, 21, 37]. The remaining studies either did not adjust for smoking or did not clearly report such adjustments, often due to reliance on self-reported data or lack of detailed covariate control. This variability likely contributes to the observed heterogeneity across outcomes. While we considered stratifying our analysis by adjustment status, the inconsistent reporting of adjustment methods and the lack of standardized data on smoking adjustment across studies precluded a meaningful meta-analytic separation. Instead, we relied on the random-effects model to account for this variability, ensuring our pooled estimates reflect the real-world diversity of study designs and confounder handling.

Another potential source of heterogeneity could be the timing of cannabis exposure during pregnancy, which our study did not stratify due to limited data granularity in the included studies. Early exposure during the first trimester, a period of rapid fetal organogenesis, might pose different risks compared to use later in gestation, potentially affecting outcomes like congenital anomalies or preterm delivery differently. While some studies in our review (e.g., Dodge et al., 2023; El Marroun et al., 2009) explored timing-specific effects, the majority provided only aggregate exposure data, precluding a meta-analytic stratification by trimester [29, 32]. This limitation is inherent to the retrospective nature of our source material, but it highlights an important avenue for future research.

Almost all recent systematic reviews have agreed with cannabis increasing the risk of poor neonatal outcomes, especially weight outcomes [18, 77, 81], preterm delivery [18, 77, 81], and NICU admission [18, 81]. However, secondary to the large number of included studies, this analysis was able to include many other neonatal outcomes that have not been thoroughly addressed in previous analyses. These outcomes included fetal anomalies, neonatal mortality, birth length, head circumference, and decreased gestational age. This is considered a strength of our review. Moreover, we found maternal cannabis use was associated with an increased risk of infant death during the first year of life, with an adjusted risk ratio of 1.72 compared to non-users. This findings is consistent with a 2023 retrospective study, Bandoli et al. [82], that specifically analyzed this outcome and further found that the specifically increased causes of mortality were sudden unexpected death and death attributable to perinatal conditions.

Many recent studies have also supported the association of cannabis consumption with anomalies affecting many systems like gastrointestinal, neuronal, nephrological, cardiovascular and musculoskeletal, although there is no consensus as to what the mechanism of this damage truly is [83–86]. Some authors have hypothesized that this may be secondary to cannabis’s role in the methylation of fetal DNA, which may increase the risk of birth defects and other anomalies [87]. Others have postulated that it could be cannabis’s role in glucose and insulin regulation that affects fetal growth and may explain its teratogenicity [32]. As the endocannabinoid system is important in the early stages of cell survival and formation of the neuronal system [88], other authors have suspected that disruption of this system may be the cause of birth defects and other adverse neonatal outcomes associated with cannabis [89]. Lastly, other authors have speculated that cannabis damages placental endocrine function by enhancing ESR1 and CYP19 AI transcription, which may increase estradiol production, causing disruption [90].

Besides neonatal outcomes, the association between cannabis use and maternal complications is also controversial. Many studies have found pregnant cannabis users were found to have higher risks of less studied outcomes not included in this study, including alcohol consumption, anemia, depression, and anxiety [16, 50, 91]. However, when focusing on the most commonly studied outcomes, such as placental abruption, antepartum or postpartum bleeding, and gestational hypertension, most [50, 62, 91], but not all [92] studies showed no significant association with cannabis use. Lastly, we found an unexpected result compared to the previous literature on the decreased risk of gestational diabetes mellitus in cannabis users compared to non-users. Most previous studies have found no association [50, 62, 91], and one study, Porr et al. [93], actually found that cannabis use was associated with increased HbA1c in diabetes mellitus. Another study, Ayonrinde et al. [94], also found that cannabis use increased the caloric intake, weight, and percentage of fatty liver during pregnancy, which in turn increased insulin resistance. Pan et al. [95] in 2023 found that preconceptional cannabis use was associated with increased gestational diabetes risk in pregnant women who never used tobacco; however, among those on current or previous using tobacco, no significant results were observed. Consistent with these studies and as stated above, we believe the protective association we have seen against gestational diabetes is most likely not a true signal, and is secondary to the likely higher percentage of pregestational diabetics in the cannabis use group, making it impossible for these women to receive a diagnosis of gestational diabetes during pregnancy. Unfortunately we do not have the specific data as to the percentages of pre-existing diabetics in both groups that would be necessary to test this hypothesis.

### Strengths and limitations

Our primary strength lies in the inclusion of 7,920,383 women, making this the largest meta-analysis to date on cannabis use in pregnancy, and our examination of a broad range of maternal and neonatal outcomes, many of which were underexplored in prior reviews. However, we recognize several limitations, notably the significant heterogeneity across studies, which is inevitable given the scale and diversity of our 51 included studies. Key sources of this heterogeneity include concomitant tobacco smoking, variations in exposure timing, cannabis consumption methods (e.g., smoking vs. ingestion), and concurrent use of other substances like alcohol. Specifically, while Table 2 provides raw numbers of smokers in each study, only about 39% of studies (20/51) explicitly adjusted for smoking in their analyses, as reviewed from their methodologies (e.g., [15, 21, 37]). This inconsistency in confounder adjustment, particularly for smoking—a known risk factor for adverse neonatal outcomes—may influence our pooled estimates. Additionally, our binary classification of cannabis use (users vs. non-users) may obscure nuances in frequency, quantity, and recency of use, while varied exposure ascertainment methods (Table 1) add further complexity. However, re-analyzing the data to separate studies by smoking adjustment status was not feasible due to incomplete or unclear reporting of adjustment methods in many studies, which would compromise the validity of such subgroup analyses. Our use of the random-effects model mitigates this by accounting for such variability, and reliance on observational data inherently increases bias risk, including from self-reported cannabis use. We recommend future studies standardize confounder reporting, particularly for smoking, to enable more precise analyses, but believe our current approach maximizes inclusivity and generalizability without necessitating additional stratification.

## Conclusion

Cannabis use is associated with adverse neonatal outcomes including low birth weight, small for gestational age, major anomalies, decreased head circumference, decreased neonatal weight, decreased birth length, decreased gestational age at time of delivery, higher rates of NICU admissions, higher rates of perinatal mortality, and a higher rate of preterm delivery. We also found that cannabis use was associated with decreased risk of gestational diabetes, although we are cautious about overinterpreting this finding and believe it may be related to cannabis users having a higher rate of pregestational diabetes. We believe that the size of this study can help bring consensus to the debate of cannabis’s associate with adverse neonatal outcomes, and would very much like to see more prospective observational studies, especially those classifying patients according to the concomitant use of tobacco products and by the different different delivery methods of cannabis products. While variability in smoking adjustment across studies limits our ability to isolate its confounding effects fully, the large sample size and consistent associations strengthen the clinical implications of these findings. Future research with uniform adjustment for confounders like smoking could refine these estimates, but our current results robustly support counseling against cannabis use in pregnancy.

## Data Availability

No datasets were generated or analysed during the current study.
